# Hospital and Institutional News

**Published:** 1920-10-09

**Authors:** 


### HOSPITAL AND INSTITUTIONAL NEWS.

### RED CROSS SCHEME OF DISTRIBUTION.

Sir Arthur Stanley, speaking at a recent luncheon at the Automobile Club, said that all money collected during Red Cross Week will be spent on work at home, and 75 per cent. of the money collected in any one county will be retained for that county, only the remainder going to the Headquarters Central Fund. The County organisations have full discretion in spending their 75 per cent. on their various schemes of public health services. In some counties Red Cross grants have already been made for the provision of hospital accommodation, on condition that a certain sum was raised locally. The Red Cross Week and " Our Day " appeal will provide the required money, and each county concerned, he hopes, will be enabled to claim the grant. The application of money will vary very considerably. Among the poor and working classes in a large city it is estimated that 90 per cent. of the sick are cared for at home, and the aim of the Red Cross is that there should be a visiting nurse in every community. Another subject in which it is desired to interest the counties is the provision of convalescent homes. If there were a larger number of such homes at the seaside, or in healthy country districts, many persons in the earliest phases of disease might be so built up as not to become hospital patients. The 25 per cent. contribution to headquarters is intended to supplement the funds raised by some of the poorer counties and to carry on educational propaganda in regard to public health services, concentrating rather on preventive measures.

### "OUR DAY" EVENTS.

On " Our Day " street collections will be held throughout Greater London, a charity *matinée* will be given at the London Pavilion and a dance at the London Country Club, Hendon. A produce market will be organised at Devonshire House by Mrs. McKenna, and Major-General Sir R. Ewart will provide a concert at the Central Hall, Westminster, for about 1,000 wounded soldiers. The Royal Artillery band will give a performance at the Royal Artillery Theatre, Woolwich, and bazaars, whist drives, and dances are being organised in many of the London boroughs. Collections are being arranged on other days during Red Cross week—at football matches, theatres, music-halls, and cinemas. Miss Clarke Lloyd is organising a dance and variety entertainment to be given at Kensington Town Hall on October 13. The proceeds of the Victory Ball at the Albert Hall on November 11 will be equally divided between the Jubilee Nurses' Fund and the Joint Council of the British Red Cross Society and the Order of St. John.

### THE BRITISH HOSPITALS ASSOCIATION.

The next of the series of meetings to form regional committees of the British Hospitals Association will, by kind permission of the Governors, be held at the General Hospital, Nottingham, on Thursday, October 21, at 3 p.m. Mr. H. Wade Deacon, the Chairman of the British Hospitals Association and of the Royal Infirmary, Liverpool, will be the principal speaker. Copies of the important address delivered by Sir Napier Burnett at the British Hospitals Association meetings at Birmingham and Manchester (see The Hospital of September 25, page 652) will reach members probably early next week. Should additional copies be required they can be obtained at the new offices of the Association, which are at 33 Bloomsbury Square, where any future business communications should be addressed.

### A PENNY PER WEEK PER POUND.

The members of the Yorkshire Voluntary Charitable Associations at their recent annual meeting at Wilsden, Yorks, heard a strong defence of the voluntary hospitals from Mr. Henry Pennington, of Bradford, the city which, as he reminded his audience, has decided to be the first to have its municipal hospital. After remarking that the cost of that plan would be £62,000 a year or an average increase in rates of 45s. a year on a £15 house, he dealt with an alternative. This was for the workers to agree to contribute one penny per week per pound earned. The sum so realised would suffice to maintain the four principal hospitals in Bradford. A campaign in favour of this scheme has been begun, and if it succeeds the financial problem will be solved. To maintain the hospitals municipally will cost twice as much and the work will be done half as well; for sympathy is a mark of voluntaryism just as interference and red tape are marks of public bodies. The penny per pound plan has a good deal in its favour. It is a fixed rate which can automatically follow the ups and downs of wages; and in view of their general increase corresponds more or less to the proportion which the old penny per week represented before the war. We hope, therefore, that the principle will find general acceptance and that other cities will follow Bradford in its advocacy.

We suppose that Mr. Pennington has a responsible authority to quote for his figures, but we cannot understand how the Municipal Hospital is going to put 3s. a £ on the rates of Bradford, however extravagantly it is managed. Under the figures quoted the rateable value of Bradford works out about £400,000, or only just a little more than one of the smaller boroughs of London. But the annual cost as stated, which is the basis of our calculation, is impossibly low. Sir William Collins, in a recent interview, said that according to the Poor-Law Commission there already exists the power to establish and maintain hospitals out of the rates for any class of disease or patient. But Dr. Addison, when asked in the House of Commons on this very point, was far from confident that municipal authorities had this power.

### DIAGNOSTIC TESTS FOR TUBERCLE.

In a recent issue of the *Lancet* we note that a contributor has published the result of some experiments carried out under the guidance of the Medical

Research Council, in which it is attempted to prove that a really reliable complement fixation test (comparable with the Wassermann reaction), can be established for the diagnosis of early tubercle. At present it is a preliminary report only, but if this work stands the test of investigation in other hands and in general practice, there is no doubt that a valuable step forward will have been made. While we reserve our judgment on the actual possibilities of yet another specific laboratory test, it is interesting to note the comments which our correspondent makes on page 28. Laboratory work on tuberculosis has produced so many disappointments as well as triumphs in the past.

### MR. WYNNE BAXTER.

We regret to read of the death of Mr. Wynne Edwin Baxter, the well-known London coroner, who died on October 1 at Stoke Newington. He served for thirty-three years as coroner for East London and the Tower of London, and continued in office until his illness about three weeks ago. Born at Lewes in 1844, the son of the proprietor and editor of the *Sussex Express*, Mr Baxter was admitted a solicitor in 1867, and appointed coroner for Sussex in 1876. He conducted the inquiry into the murder of Mr. Gold, a retired merchant, who was shot while travelling by a Brighton express in June 1881; and held the inquest on Lefroy, who was executed for the crime in November of the same year at Lewes Gaol. Mr. Baxter was appointed coroner for East London in 1887, and among the first inquests held by him were those on the victims of the mysterious "Jack the Ripper." He also held the inquests on the policemen who were shot by Russian anarchists at Houndsditch in January 1911, and on the bodies of the criminals themselves who were killed in the Sydney Street affair. After the Silvertown explosion during the war Mr. Baxter held, on one day alone, inquests on over sixty bodies at the London Hospital, on which occasion not only were the ordinary mortuaries filled, but bodies had to be laid also in rows along the corridors. As Coroner of the Tower he held inquests on the bodies of the German spies who were condemned to death by courts-martial and shot. He held, on an average, 1,000 inquests annually. One year the number was 2,500.

### THE ANATOMY ACTS.

The Minister of Health has appointed Dr. Alexander Macphail to be a Medical Officer of the Ministry. Dr. Macphail was formerly professor of anatomy in St. Mungo's College, Glasgow, lecturer in anatomy at St. Bartholomew's Hospital, and secretary of the Anatomical Supplies Committee of Great Britain. His first duties in the Ministry of Health will be in connection with the administration of the Anatomy Acts, which has been transferred to the Ministry from the Home Office.

### THE INFECTIOUS FEVER INCREASE.

It is a distressing and undeniable fact that the infectious fever returns show an increase in the Metropolitan area. Coincidentally arises the natural outcry that hospital accommodation for them is not everywhere as ample and satisfactory as it should be. But it is unnecessary that the popular press should unduly alarm the public as to this annually recurring condition. The recent big increase is remarkable chiefly for the fact that it has taken place earlier than was either anticipated or is usual according to the seasonal incidence of these fevers. In reality, the figures are not excessive when compared with those of the pre-war years. The great and really only point which the public need remember is that the present is one of the infectious fever seasons, and that all parents have the need for corresponding watchfulness imposed upon them.

### MATERNITY AND WELFARE.

It is with the greatest satisfaction that we see the public attention being drawn more and more insistently to the problems, and, indeed, to the great activities, which are everywhere taking place in connection with maternity and child welfare. The development, financing, and voluntary support of the "centre" principle are receiving deserved prominence. This week we have the teachings before us which arise from the Maternal Birth-rate Commission discussions. But there is, running in a perfect parallel with these matters, the question of that great danger to the recently delivered mother, namely the incidence of puerperal sepsis. The subject is one which has been frequently ventilated in these columns, but the frequency of and the mortality associated with this avoidable catastrophe are such that the further review of the matter which our contributor makes on page 27 is more than justified.

### WOMEN IN MEDICINE.

Some months ago we expressed our agreement with the statements made by several authorities in the world of the medical schools that due consideration should be given to the future outlook by a woman considering the advisability of now entering the medical profession. There are, indeed, more intricate difficulties abroad than simply those which arise with overcrowding. For the moment it is not really surprising to read in the pages of a contemporary the statement that at present the available posts are more numerous than the women doctors who want them. But this situation must not be taken as of a necessarily permanent nature. In fact, it appears to us likely that the time is fast approaching when all but the best of the women doctors will have no little difficulty in finding a perfect array of posts at £600—our contemporary's figures—from which they can choose at their leisure.

### ALEXANDRA DAY.

The administrative committee of the Alexandra Day Fund met at the Mansion House on September 30, when a message from Queen Alexandra was read expressing thanks to all workers and contributors. A sum of £31,155 collected in London was distributed amongst institutions and charities. Of this £9,855 was allocated by Queen Alexandra to institutions and charities in which she takes especial interest, and the remaining £21,300 was dispersed in accordance with the recommendations of the Mayors and local committees in the various districts where collections were made.

### THE MISCELLANEOUS PROVISIONS BILL.

We understand that political opposition to Dr. Addison's proposal to assist hospitals out of local rates is so great that the Government will probably drop this part of the Miscellaneous Provisions Bill. There is a rumour that Dr. Addison's original idea was more in the direction of State aid, which would be much preferred by the hospitals themselves. It is stated that only by Treasury opposition a grant of £500,000 from the Exchequer failed to materialise. The ratepayers themselves are getting alarmed at the terrific increase in the claims upon them, and if the burden of local taxation is to be further increased, and the municipality given such very wide powers to spend money on hospitals, we are not surprised at the disquietude of the middle-class citizen.

### THE PLANS OF THE NEW INFIRMARY AT PETERBOROUGH.

The open competition for the plans for a new war memorial at Peterborough led to the submission of twenty-nine designs, which have been exhibited at the old Free Library. Mr. Edwin T. Hall, F.R.I.B.A., was the assessor, and the successful competitors were: First prize (£200): Mr. Wallace Marchment, 41 Ovington Street, Cadogan Square, S.W.; estimated cost, £80,822. Second prize (£100): Messrs. Kenyon & Livock and Messrs. Aylwin & Bowring, 26 Great St. James Street, Bedford Row; estimated cost, £97,056. Third prize (£50): Mr. Horace Cubitt, 7 New Square, Carey Street, W.C.; estimated cost, £112,000. It will be noticed that the most economical design is that which has been placed first. It provides for 122 beds, at a cost of nearly £700 per bed.

### A COSTLY FETE AT STOURBRIDGE.

A word of explanation seems to be required in regard to the proceeds of the fêtes organised by the Fêtes Committee for the benefit of Corbett Hospital, Stourbridge. The Committee is reported to have had a record year, and to have received during the two days £1,564. But the expenses incurred are stated to have been no less than £1,140, so that the balance was only £524. In other words, some 60 per cent. was eaten up by expenses. What is the explanation? The figures are sufficient to make one desirable, but since Dr. H. D'Arcy Ellis, the medical officer, has reported the hospital to be overcrowded, and there is urgent need for extension, the high cost of the recent effort is doubly regrettable.

### A HOSPITAL AND EJECTED TENANTS.

It appears from a case in the Borough Police Court that the 1st Eastern General Hospital at Cambridge is being used residentially by those who cannot find houses. The applicants are very numerous, far more numerous than the accommodation can house. A small committee investigates applications and preference is given to ex-soldiers, then to others in order of their deserts and the urgency of the case. Mr. J. P. Bland, who is in charge of the Borough Housing Scheme, calls attention to the excess of applicants, because landlords who wish to regain possession of their houses incline to allege that accommodation can be found by tenants at the 1st Eastern General Hospital.

### HOLMESDALE COTTAGE HOSPITAL.

In resigning the presidency of Holmesdale Cottage Hospital, Sevenoaks, which he has held for the past ten years, Earl Stanhope calls attention to his three unsuccessful attempts to raise sufficient funds to extend the institution. He resents the criticisms made against the committee for not altering the rules so as to admit other than weekly wage-earners, and points out that the allegation of vacant beds which residents of a different class could conveniently use is unfounded, because vacancies must be kept for accidents and emergencies. The committee, he says, is aware how insufficient the hospital is for the twenty thousand inhabitants of the district, and that extension is imperative, but that temporary huts only can be added at present. Sevenoaks, says Earl Stanhope, has no right to be proud of its inability or unwillingness to provide an adequate hospital. He therefore resigns the task to his successor, because he has no reason to suppose that a fourth attempt on his part would be successful. It seems clear that no one will readily accept the task unless there is assured support from the district. We hope that the reason for Earl Stanhope's resignation will bring home to the district the reproach of which he speaks.

### BIRMINGHAM HOSPITALS' LEAGUE.

A public meeting has been held at Birmingham to inaugurate a Hospital League to find additional sources of income. Mr. C. H. Saunders, chairman of the board of the general hospital, presided, and Mr. A. E. Jowett recalled that the plan to establish maintenance charges for patients had proved so unpopular that its consideration had been postponed to see if the League could raise the necessary money. It was eventually decided to invite the board of the general hospital to include all the Birmingham hospitals wishing to join in the scope of the League, which has already received £1,050. The discussion chiefly concerned the desirability of making the League serve all the hospitals, though the idea originated with the general hospital. Little was said about the organisation of the League or of its particular plan of raising money. It will, however, be distinct from any other body or hospital fund, and if it organises subscriptions from those not associated with the other funds it will have certainly justified itself.

### HOSPITALS IN LEICESTER.

According to Dr. C. Killick Millard, the Medical Officer of Health, Leicester, is reasonably supplied with hospital accommodation. There is an excellent general hospital, the Leicester and Leicester shire Royal Infirmary, providing some 300 beds, including a children's block of seventy beds. The working classes—through the Saturday Fund, which makes weekly collections at most of the factories—contribute many thousands of pounds per annum towards the upkeep of the Royal Infirmary, and also, through the same fund, maintain their own convalescent homes. There is a much smaller semi-private hospital, the Faire Hospital, where for a reasonable payment persons rather above the working classes can obtain treatment and have operations performed. There are now two maternity hospitals in the city. One is supported by voluntary contributions supplemented by payments for admission, provides twenty beds, and has been in existence for fifteen years. The other was opened at the end of 1919 by the corporation. The guardians have an excellent modern Poor-law infirmary, providing 550 beds, including a detached lying-in block.

### APPEALS PLUS CONTRIBUTIONS.

The Saturday Funds, particularly in certain districts—like Manchester, for instance—are showing an excellent spirit, not merely in the care and generosity with which their contributions are raised, but also in the additional issue of appeals. They organise these with the help, now more often given, of employers, by whose leave collecting sheets are placed in the different departments of the firm, with explanatory leaflets, and in Manchester the Saturday Fund has organised another hospital week. The scheme is indeed comprehensive, and included collections in the picture houses, on the City football ground, and, in fact, to all and sundry. Last time over £6,000 was raised by the Saturday Fund appeal, and it is hoped to do much better, for the sum is a small one for Manchester. We now see hospitals seeking weekly contributions where they used to seek subscriptions, and the funds which invented the weekly contribution following these up by general appeals. This shows a general awakening in all interested in the voluntary system, and is another instance of co-ordination and mutual aid. The co-ordination is far from complete, but it is growing, and there is reason to hope that the voluntary system may, once again, save the situation.

### COLONEL D. J. MACKINTOSH AT CARDIFF.

The Committee of King Edward VII. Hospital, Cardiff, has now received an interim report from Colonel D. J. Mackintosh, of the Western Infirmary, Glasgow, who was asked to advise on the structural development of the hospital. Judgment in respect of the report has been suspended till finance involved and the general financial prospects have been considered. But quietly Dr. Mackintosh recommended that, while the nurses' quarters might be rearranged, no further structural alterations should be attempted until a complete scheme of the needs of the area in relation to the University Medical School has been prepared We understand that four-fifths of the appeal for £150,000 have been given or promised, but the overdraft and the waiting-list are still distressingly large.

### THE EXTENSIONS AT NOTTINGHAM.

Sir Jesse Boot's recent gift of £50,000 to maintain the Cedars and Woodthorpe Lodge Convalescent Homes, associated with Nottingham General Hospital, has led the convalescent hospital committee to see if both homes cannot be made more useful by admitting cases from the general hospital earlier than at present. The structural alterations involved, including accommodation for night nurses, Sir Jesse Boot has also generously agreed to provide. Meantime, in regard to the War Memorial, a new nurses' home is to be built without delay, and as soon as funds permit, the provision of further beds will be begun. Sir Jesse Boot's gift, with the committee's new proposal, will do something as it is to relieve the pressure, and should form an earnest of the memorial extension scheme.

### ASSISTANCE FROM "THE TRADE."

The Royal National Orthopædic Hospital, which needs £200,000 to cope with its work and long list of waiting cases, has received the aid of the *Shoe and Leather News*, which is seeking support from the trade for the hospital. The needs are for more beds, for a country home, a school of orthopædics, and means to use the adjacent side already acquired. The heads of the boot-manufacturing industry are supporting the Editor's appeal, and it is hoped to arrange an organised effort among the retailers. We hope that their *esprit de corps* will serve the London hospital in which they can take a most professional interest.

### MANSIONS AS SANATORIA.

The tendency of the times is to buy old mansions for sanatoria instead of building new ones. The Herefordshire County Council, which purchased an eighty-six-acre site at Willian for a county sanatorium in 1915, now finds that to provide for 220 patients and staff would cost £121,000. The Council, therefore—with the consent of the Ministry of Health—proposes to buy Ware Park mansion and grounds, comprising in all 112 acres, and by means of huts to provide accommodation for 132 patients at a total cost of £64,000. The Health Ministry's grant will be £180 per bed.

### THIS WEEK'S DRUG MARKET.

Quiet conditions continue to prevail in the drug market, and such buying as goes on is restricted to small quantities. In some instances, the drop in prices which has been going on gradually for so long a period has, in the aggregate, been very considerable, and the margin, in such cases, between the cost of production and the normal market value is so narrow that further reductions could only be made at a loss. The number of such instances, however, is limited, but the fact is mentioned as a reminder that there must come an end to price recessions sooner or later. Among the articles which have again fallen in value are potassium bromide, sugar of milk, citric acid, tartaric acid, cream of tartar, codeine, honey, ergot of rye, hexamine, ipecacuanha and balsam tolu. There is still a good inquiry for eucalyptus oil, the price of which is firmly maintained.

